# Real-Time Imaging of Short-Wave Infrared Luminescence Lifetimes for Anti-counterfeiting Applications

**DOI:** 10.3389/fchem.2021.659553

**Published:** 2021-04-26

**Authors:** Roman Ziniuk, Artem Yakovliev, Hui Li, Guanying Chen, Junle Qu, Tymish Y. Ohulchanskyy

**Affiliations:** ^1^Key Laboratory of Optoelectronic Devices and Systems, Center for Biomedical Photonics and College of Physics and Optoelectronic Engineering, Shenzhen University, Shenzhen, China; ^2^School of Chemistry and Chemical Engineering, Harbin Institute of Technology, Harbin, China

**Keywords:** photoluminescence, time-gated imaging, short-wave infrared, rare-earth ions, nanoparticles, anti-counterfeiting, rapid lifetime determination

## Abstract

Rare-earth doped nanoparticles (RENPs) have been widely used for anti-counterfeiting and security applications due to their light frequency conversion features: they are excited at one wavelength, and they display spectrally narrow and distinguished luminescence peaks either at shorter wavelengths (i.e., frequency/energy upconversion) or at longer wavelengths (frequency/energy downconversion). RENPs with a downconversion (DC) photoluminescence (PL) in short-wave infrared (SWIR) spectral range (~1,000–1,700 nm) have recently been introduced to anti-counterfeiting applications, allowing for multilevel protection based on PL imaging through opaque layers, due to a lesser scattering of SWIR PL emission. However, as the number and spectral positions of the discrete PL bands exhibited by rare-earth ions are well-known, it is feasible to replicate luminescence spectra from RENPs, which results in a limited anti-counterfeiting security. Alternatively, lifetime of PL from RENPs can be used for encoding, as it can be finely tuned in broad temporal range (i.e., from microseconds to milliseconds) by varying type of dopants and their content in RENPs, along with the nanoparticle morphology and size. Nevertheless, the current approach to decoding and imaging the RENP luminescence lifetimes requires multiple steps and is highly time-consuming, precluding practical applications of PL lifetime encoding for anti-counterfeiting. Herein, we report the use of a rapid lifetime determination (RLD) technique to overcome this issue and introduce real-time imaging of SWIR PL lifetime for anti-counterfeiting applications. NaYF_4_:20% Yb, x% Er (x = 0, 2, 20, 80)@NaYF_4_ core@shell RENPs were synthesized and characterized, revealing DC PL in SWIR region, with maximum at ~1,530 nm and PL lifetimes ranging from 3.2 to 6 ms. Imaging of the nanoparticles with different lifetimes was performed by the developed time-gated imaging system engaging RLD method and the precise manipulation of the delay between the excitation pulses and camera gating windows. Moreover, it is shown that imaging and decrypting can be performed at a high rate (3–4 fps) in a cyclic manner, thus allowing for real-time temporal decoding. We believe that the demonstrated RLD-based fast PL lifetime imaging approach can be employed in other applications of photoluminescent RENPs.

## Introduction

The forgery and counterfeiting of all kinds have become a real threat to a number of industries including banking, pharmaceuticals, electronics, and packaging (Chaudhrya et al., 2005; Fincham, 2014; Fink et al., 2016). To protect and ensure legitimacy of valuable commodities, various protection techniques were introduced, including watermarking, laser holography, radio-frequency identification, and luminescent anti-fake patterns. Luminescence-based anti-counterfeiting strategy has attracted attention due to advantages of good concealment, difficulty of faking, low production cost, and feasibility of large-scale production. Luminescent materials can be used to create protective tags, labels, or packages; unique patterns [e.g., barcodes or quick response (QR) codes] can be created, revealing encoded information either under photoexcitation (with light at certain wavelength or by natural day/room light) or under specific external stimuli (e.g., pressure) (Kumar et al., 2014; Liu et al., 2017; Sun et al., 2017; Han et al., 2019; Abdollahi et al., 2020). Optical properties of those materials (luminescence spectra, lifetime, etc.) play a crucial role in optical information encoding and determine properties of the security patterns.

Rare-earth ion doped nanoparticles (RENPs) are well-known and widely used due to their light conversion feature that leads to the spectral fingerprint of their photoluminescence (PL), distinguishable by large Stokes (downconversion, DC) and anti-Stokes (upconversion, UC) shifts (Liu et al., 2011; Andres et al., 2014; Kaczmarek et al., 2017; Mei et al., 2019; Xie et al., 2019; You et al., 2019). RENPs provide a number of unique features for anti-counterfeiting and security applications, such as a color-tunable UC/DC luminescence with long decay time and excellent stability. In addition, RENPs can feasibly be introduced into polymeric/polymerizable materials for UC/DC PL pattern fabrication or transferred to the ink-like solutions compatible with commercially available inkjet printers for further pattern printing (da Luz et al., 2015; You et al., 2015; Jee et al., 2020).

Among all the RENPs, Yb^3+^/Er^3+^ doped UC nanoparticles have been widely recognized, extensively studied, and used in various applications due to their bright UC/DC emission. Being excited in the intense absorption peak at ~980 nm, Yb^3+^ sensitizes Er^3+^, which manifest UC PL in visible spectral range, as well as DC PL peaked at ~1,530 nm (Yi et al., 2004; Li et al., 2017; Secu et al., 2019). It should be noted that RENPs with DC PL in short-wave infrared (SWIR) spectral range are increasingly employed in various imaging applications, due to lesser scattering. SWIR light can penetrate deeper through opaque media in comparison with visible and near-infrared one (Bashkatov et al., 2005; Laurenzis and Christnacher, 2013; Göhler and Lutzmann, 2016; Golovynskyi et al., 2018). SWIR is usually defined by the hardware manufacturers as ~1,000–1,700 nm spectral range, which is associated with a range of sensitivity of InGaAs detectors and, correspondingly, InGaAs-based imaging cameras. At the same time, the SWIR term specifically defines the spectral range beyond 1,500 nm (Golovynskyi et al., 2018), especially in rapidly emerging bioimaging field that utilizes exogenous photoluminescent probes, including Yb^3+^/Er^3+^ doped RENPs (Wang et al., 2018a; Lv et al., 2019; Hazra et al., 2020; Li et al., 2020). Along with this, anti-counterfeiting applications of the SWIR emitting nanoparticles have also been recently reported, suggesting PL imaging of 3D patterns and multilevel anti-counterfeiting protection based on imaging through opaque layers (Tan et al., 2020; Platel et al., 2021).

**Figure 1:**
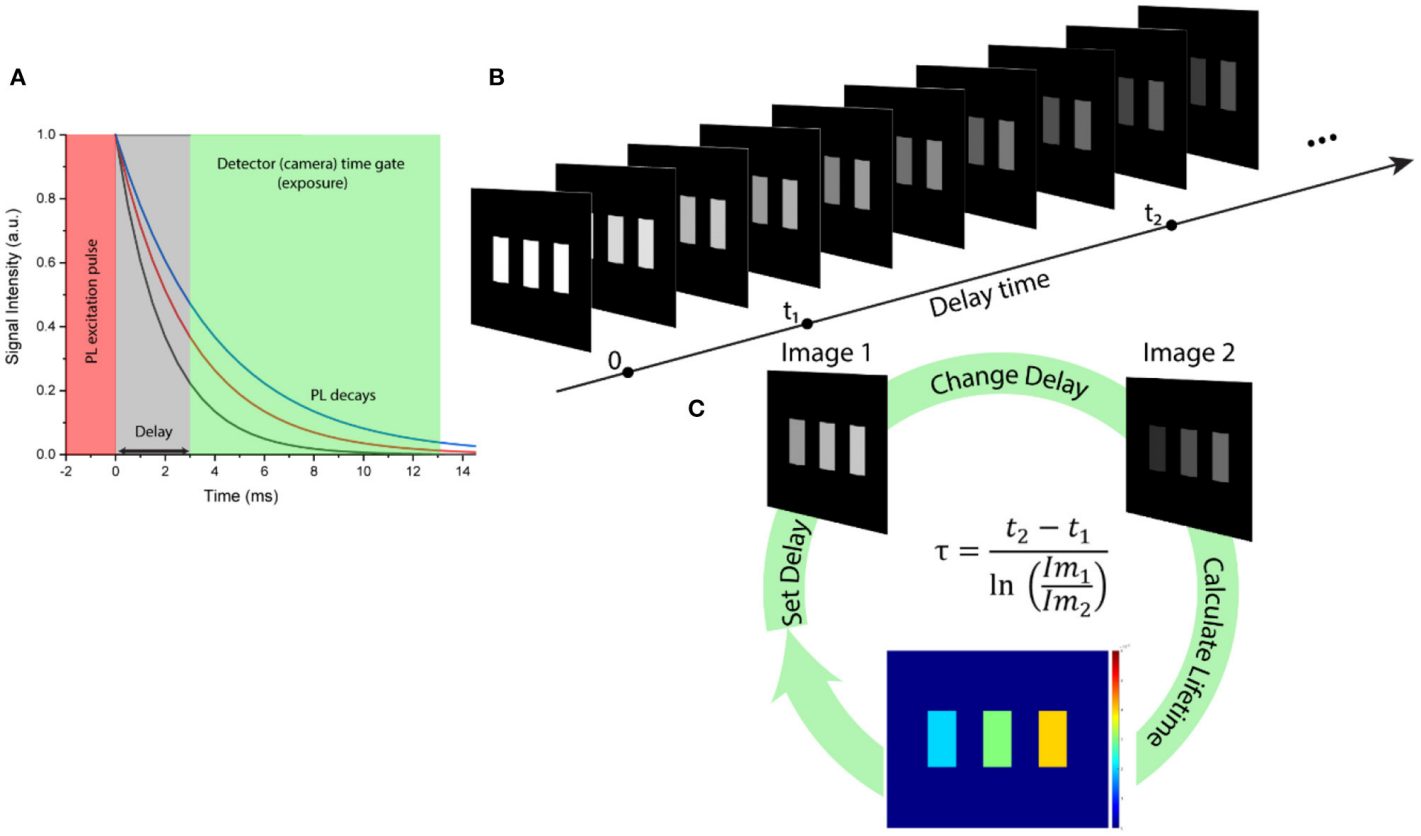
**(A)** Schematic representation of the PL signal selection using time-domain filtering. **(B)** Stack of time-gated PL images acquired with gradually increased delay time. **(C)** Three-step rapid lifetime determination scheme for real-time anti-counterfeiting imaging, where *t*_1_ and *t*_2_ are delay times for acquiring Image 1 and Image 2 with Im_1_ and Im_2_ intensity values, respectively, and τ is PL lifetime calculated for each pixel.

Despite all advantages, use of RENPs for anti-counterfeiting has some limitations. In particular, the number and spectral positions of the discrete luminescence bands provided by rare-earth ions are well-defined and known, making a replication of the luminescence spectra relatively feasible and limiting security of an anti-counterfeit coding. On the other hand, lifetime of UC/DC PL from RENPs can be finely tuned in broad temporal range (from microseconds to milliseconds) by multiple methods, including the changes of dopant types and content, along with tailoring nanoparticle morphology and size (Wang and Liu, 2008; Deng et al., 2015; Fan, 2018; Ortgies et al., 2018; Wang et al., 2018b, 2019; Liang et al., 2019; Mei et al., 2019). This provides much broader range of possibilities for anti-counterfeiting patterns in comparison with the encoding based on PL spectra. The PL lifetime encoding dimension has recently been demonstrated for the anti-counterfeiting applications, exploiting time-domain PL lifetime measurement and time-gated imaging of anti-counterfeiting patterns created with SWIR emitting RENPs (Tan et al., 2020). At the same time, a decrypting of PL lifetime encoded information, as well as practical use of time-gated PL imaging systems in anti-counterfeiting applications depend on the precision of time-gated imaging setup and the image acquisition/processing time. The main part of the time gated imaging method is a precise synchronization of imaging camera shutter with the excitation pulse along with an introduction of a controllable delay between the excitation pulse and start of camera exposure to distinguish the decaying PL signal (Figure 1A). Time-gated PL imaging data (i.e., sequence of PL images) can be acquired through a stepwise increase in the delay time, with sequential acquisition of PL image for every delay (Figure 1B). Next, the obtained hypercube (stack of PL images acquired with different delays) is processed, revealing the encrypted PL lifetime information (Fan, 2018; Ortgies et al., 2018; Ziniuk et al., 2019). Processing of the hypercube can involve different PL lifetime unmixing protocols, from simple monoexponential fitting to linear mixture analysis to multiexponential fitting, or even more sophisticated approaches. These lifetime unmixing methods are applied for each pixel of the images and include complicated calculations, resulting in highly time-consuming process and revealing one of the major drawbacks of using PL lifetime in anti-counterfeit coding: the inability of instant (real-time) interpretation of the encrypted information. A scanning time-gated setup with point-to-point PL decay acquisition with consequent lifetime determination was recently proposed as a powerful solution for real-time discrimination of the PL lifetimes from multiple particles (Lu et al., 2014); however, the real-time wide-field PL lifetime imaging remains a challenge. To overcome this issue, we propose to employ a rapid lifetime determination (RLD) approach. Following this approach, lifetime of a monoexponential PL decay can be calculated from as few as two points of an exponent and PL lifetime mapping can be feasibly obtained (Sharman et al., 1999; Chan et al., 2001; Liu et al., 2014):

$$\begin{array}{l} \tau =\frac{t_{2}-t_{1}}{\ln\left(\frac{Im_{1}}{Im_{2}}\;\right)}, \end{array}$$

where *t*_1_ and *t*_2_ are delay times for acquisition of Image 1 and Image 2 with Im_1_ and Im_2_ intensity values, respectively, and τ is the PL lifetime (Figure 1C). Furthermore, by simultaneously applying the RLD approach to each pixel of an image and repeating imaging process in the cyclic manner, a real-time PL lifetime imaging can be achieved. Herein, we report on applications of the RLD approach for real-time imaging of SWIR luminescence temporal codes created using RENPs doped with Yb^3+^/Er^3+^ and emitting in 1,500–1,600 nm spectral region under 975-nm pulsed excitation for anti-counterfeiting and security applications.

## Results

**Figure 2:**
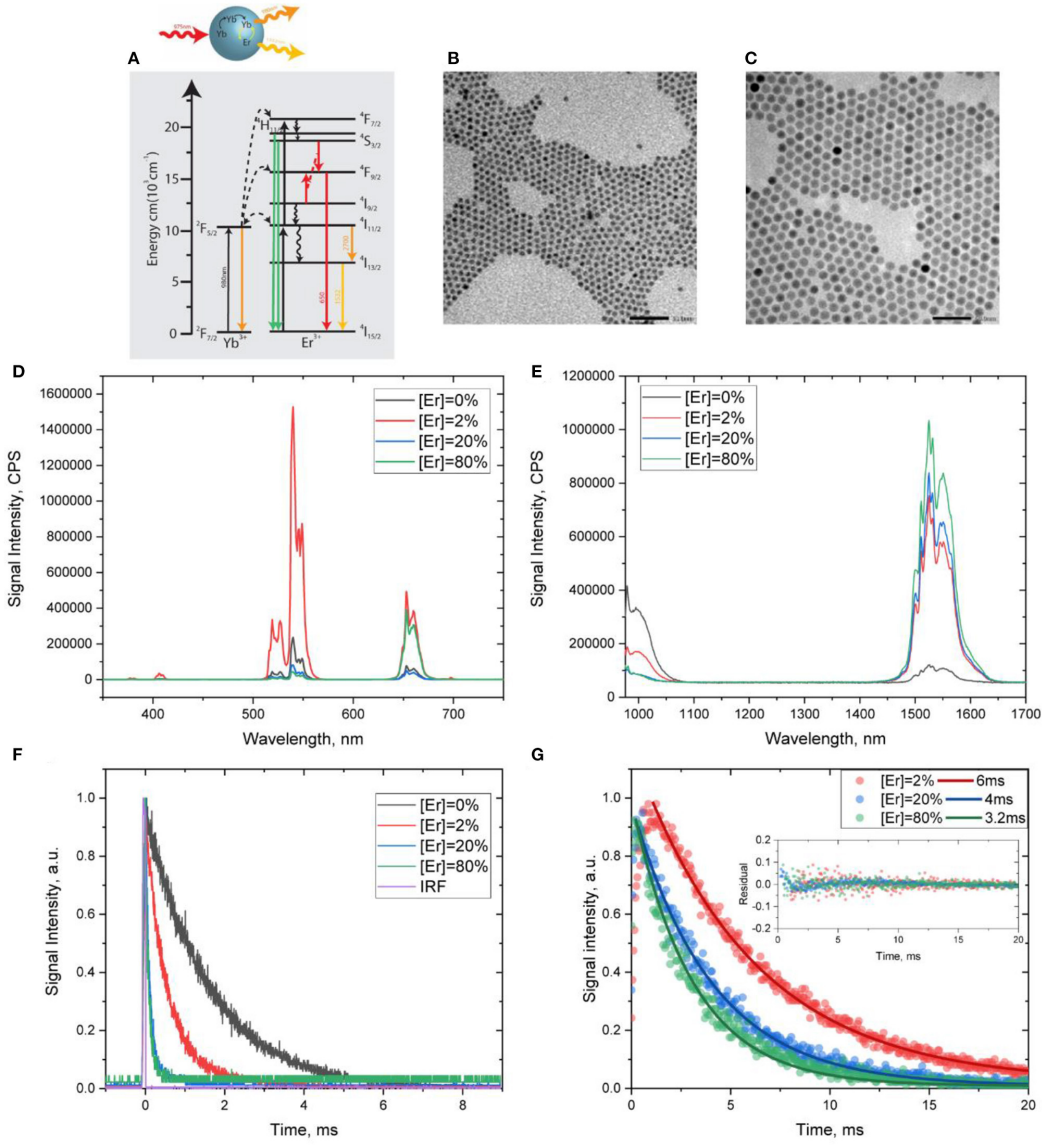
**(A)** Scheme of energy levels along with activation/deactivation pathways in Yb^3+^/Er^3+^ co-doped nanoparticles. **(B,C)** Representative TEM images of NaYF_4_: Yb^3+^, Er^3+^ core and NaYF_4_:Yb^3+^,Er^3+^@NaYF_4_ core@shell RENPs, respectively. **(D,E)** UC PL and DC PL spectra of NaYF_4_:Yb^3+^,Er^3+^@ NaYF_4_ core@shell nanoparticles under 975-nm excitation. **(F)** PL decays acquired at 1,000 nm (Yb^3+^ emission), **(G)** DC PL decays acquired at 1,530 nm (Er^3+^ emission), along with the monoexponential fits and corresponding residuals.

Core@shell RENPs of NaYF_4_:20% Yb^3+^, x% Er^3+^ (x = 0, 2, 20, 80)@NaYF_4_ were synthesized using the co-precipitation method at high temperature, as described in the Materials and Methods section. Representative transmission electron microscopy images (TEM) of the parent core NaYF_4_:Yb^3+^/Er^3+^ and core@shell NaYF_4_:Yb^3+^/Er^3+^@NaYF_4_ nanoparticles are presented in Figures 2B,C, revealing uniform size (around 5.5 and 11.5 nm, respectively) and morphology of the resulting nanoparticles.

According to the energy level diagram for Yb^3+^/Er^3+^ (Figure 2A), under excitation at 975 nm, the synthesized core@shell RENPs manifest multiphoton UC PL peaked at 650, 540, and 410 nm and originated from higher energy levels of Er^3+^ (Figure 2D). At the same time, an intense DC PL peaked at ~1,530 nm (Er^3+^ emission) is observed, along with a weaker Yb^3+^emission band at ~1,000 nm (Figure 2E). As the efficiency of excitation energy transfer between Yb^3+^ and Er^3+^ in NaYF_4_:Yb^3+^/Er^3+^ RENPs is known to depend on the dopant concentrations, an observed change in the intensities of UC/DC PL from Yb^3+^ and Er^3+^ is expected when varying Er^3+^ dopant concentrations. Indeed, when the concentration of Er^3+^ in the core was increased from 0, 2, 20, to 80%, the emission of Yb^3+^ at ~1,000 nm decreases, evidencing an increase in the efficiency of the excitation energy transfer from Yb^3+^ to Er^3+^ (Figure 2E). This was also confirmed by the shortening of the Yb^3+^ PL lifetime with an increase in Er^3+^ concentrations (Figure 2F). It is worth noting that UC/DC PL from Er^3+^ has been detected even for the RENPs that are suggested to have no Er^3+^, indicating Er^3+^ impurity of unidentified concentration, which was introduced during RENP synthesis. At the same time, as one can see in Figures 2D,E, an increase in Er^3+^ concentration from 2 to 20 and 80% resulted in a noticeable decrease in the ratio of UC and DC PL intensities. The intensity of UC PL relatively to DC PL is significantly higher for NaYF_4_:20%Yb^3+^, 2% Er^3+^@NaYF_4_ RENPs than for NaYF_4_:20%Yb^3+^, x% Er^3+^ @NaYF_4_ (x = 20, 80). This is evidently associated with the concentration quenching effect (Ding et al., 2014; Rabouw et al., 2018), which is also confirmed by the decrease in the lifetime of DC PL of Er^3+^ ions. Using monoexponential fitting, the lifetime values were found to be ~6 ms for NaYF_4_:20%Yb^3+^, 2% Er^3+^@NaYF_4_ RENPs and 4 and 3.2 ms for RENPs with 20 and 80% of Er^3+^. The DC PL decays are shown in Figure 2G along with fitting curves and residuals. It should be noted that the coefficients of determination (*R*^2^) were calculated to be 99.36, 99.35, and 99.59% for 6, 4, and 3.2 ms, respectively, confirming the accuracy of the monoexponential fitting.

**Figure 3:**
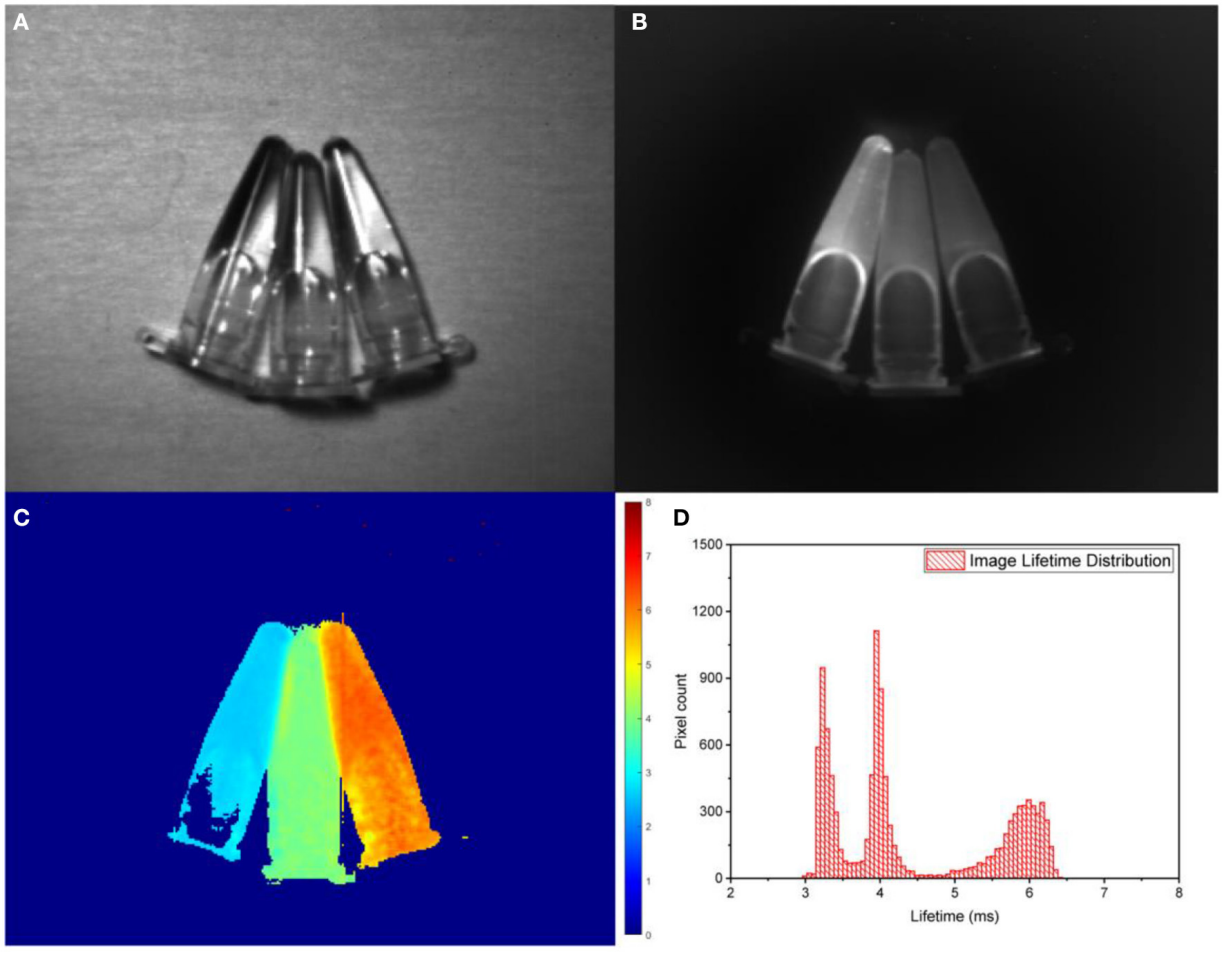
**(A)** Bright-field imaging of Eppendorf tubes with NaYF_4_:20%Yb, x%Er@NaYF_4_ (x = 2, 20, 80% right to left). **(B)** Steady-state SWIR PL imaging of Eppendorf tubes with RENPs suspensions acquired by the SWIR camera with 975-nm excitation and 1,200-nm long-pass filter. **(C,D)** PL lifetime image obtained using RLD approach and corresponding lifetime histogram.

The synthesized Er^3+^ doped core@shell nanoparticles with intense SWIR luminescence of long and tunable monoexponential lifetimes can be considered as promising constituents providing temporal codes for anti-counterfeiting patterning in scattering (opalescent) media, which is based on time-gated PL SWIR imaging (Tan et al., 2020). We have previously reported a home-built time-gated SWIR imaging system capable of collecting precisely delayed optical images of SWIR PL from RENPs excited by a pulsed laser at 975 nm (Ziniuk et al., 2019). For real-time imaging of SWIR PL lifetimes, the setup was set to acquire two PL intensity images with different delays and RLD algorithm was applied to images in order to reveal lifetimes encoded in each image pixel. To validate this concept, we imaged three Eppendorf tubes filled with suspensions of RENPs with 2, 20, and 80% of Er^3+^ and DC PL lifetimes of 6, 4, and 3.2 ms, respectively (Figures 3A,B). Utilizing two SWIR PL intensity images acquired with 1- and 5-ms delays and RLD algorithm (Figure 1), lifetime images were acquired at the rate of 4 fps, demonstrating the possibility to employ the SWIR PL lifetimes as coding (patterning) parameter in real-time anti-counterfeiting application (Figure 3C). The lifetime histogram obtained in post-imaging processing from the lifetime image file clearly visualizes the presence of three separate components in the lifetime images (Figure 3D). It is worth noting that due to the scattering of the emitted light, some area of the image (e.g., edges of Eppendorf tubes) shows different lifetimes arising from a mixture of PL signals from different samples.

**Figure 4:**
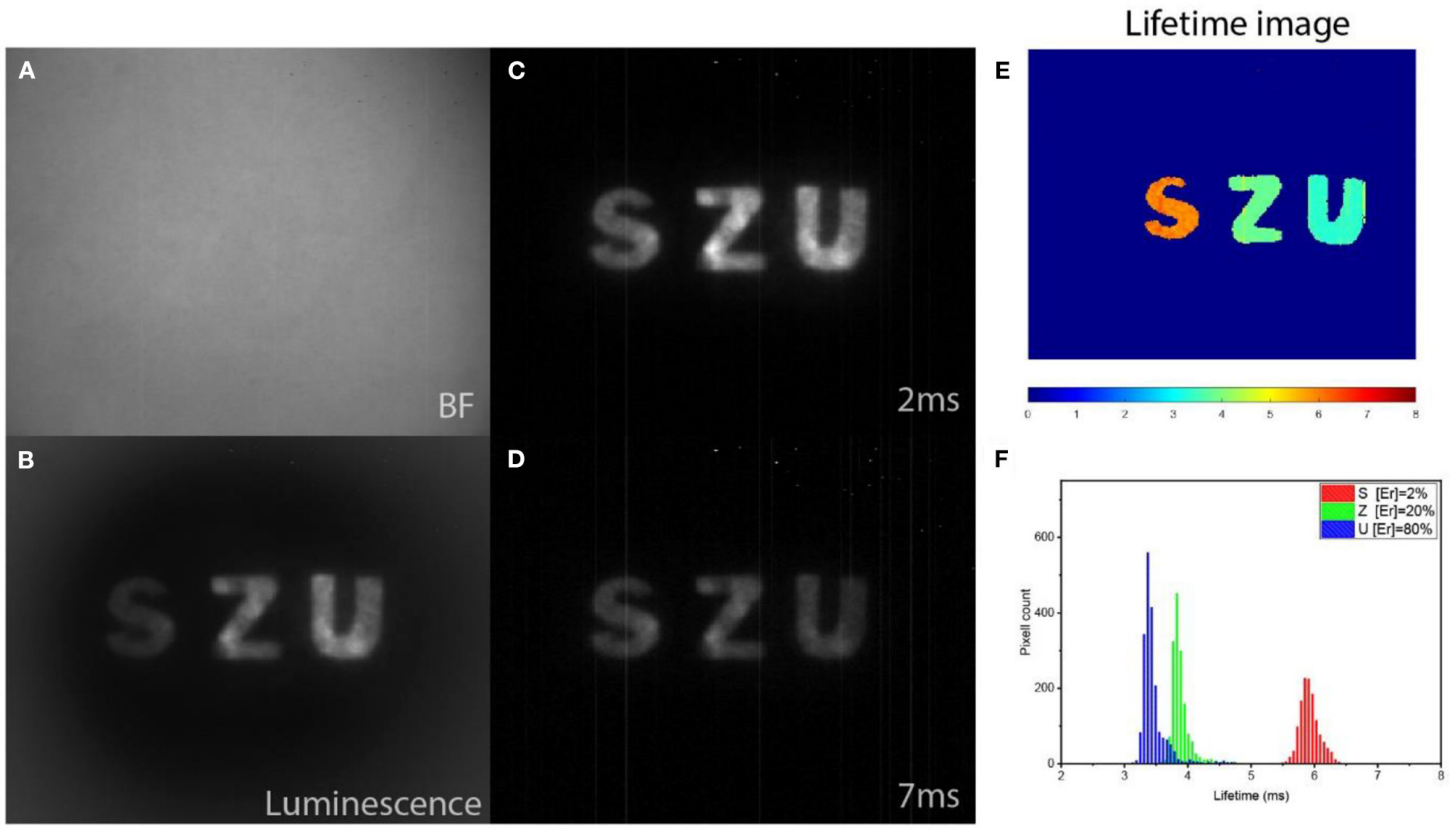
**(A)** Bright-field image of paper with invisible patterns acquired by SWIR camera at room illumination. **(B)** Steady-state PL intensity imaging revealing a SWIR PL pattern hidden under paper layer and excited by continuous wave laser irradiation at 975 nm. **(C,D)** Time-gated SWIR PL images acquired with 2- and 7-ms delays after pulsed excitation at 975 nm, respectively. **(E)** SWIR PL lifetime image generated with using RLD algorithm using images shown as **(C,D)**. **(F)** Lifetime image histogram illustrating pattern of three separate lifetime-encoded letters.

Next, a model of an anti-counterfeiting pattern was fabricated. The letters “S,” “Z,” and “U” were cut from a printing paper, soaked in the suspensions of RENPs with 2, 20, and 80% of Er^3+^ and glued between two sheets of paper. In such a way, an “SZU” pattern (Figure 4A) invisible to the naked eye was created, in which a different type of nanoparticle marked every letter. Next, steady-state and time-gated SWIR PL imaging of the pattern coded by SWIR PL lifetime were performed, using 975-nm excitation from laser diode in continuous wave (CW) and pulsed modes. When comparing steady-state and time-gated PL images, significantly improved signal-to-noise ratio for time-gated images can be clearly seen, demonstrating advantage of time-gating to cut off scattering of the excitation light (Figures 4B,C). Two time-gated SWIR PL images obtained for 2- and 7-ms delays (Figures 4C,D) were further processed by RLD-based software to determine lifetimes in each pixel (thus, revealing coded information) and produce the lifetime image shown in Figure 4E. Lifetime distribution histogram obtained from the lifetime image clearly shows encoding of each photoluminescent letter (Figure 4F).

**Figure 5:**
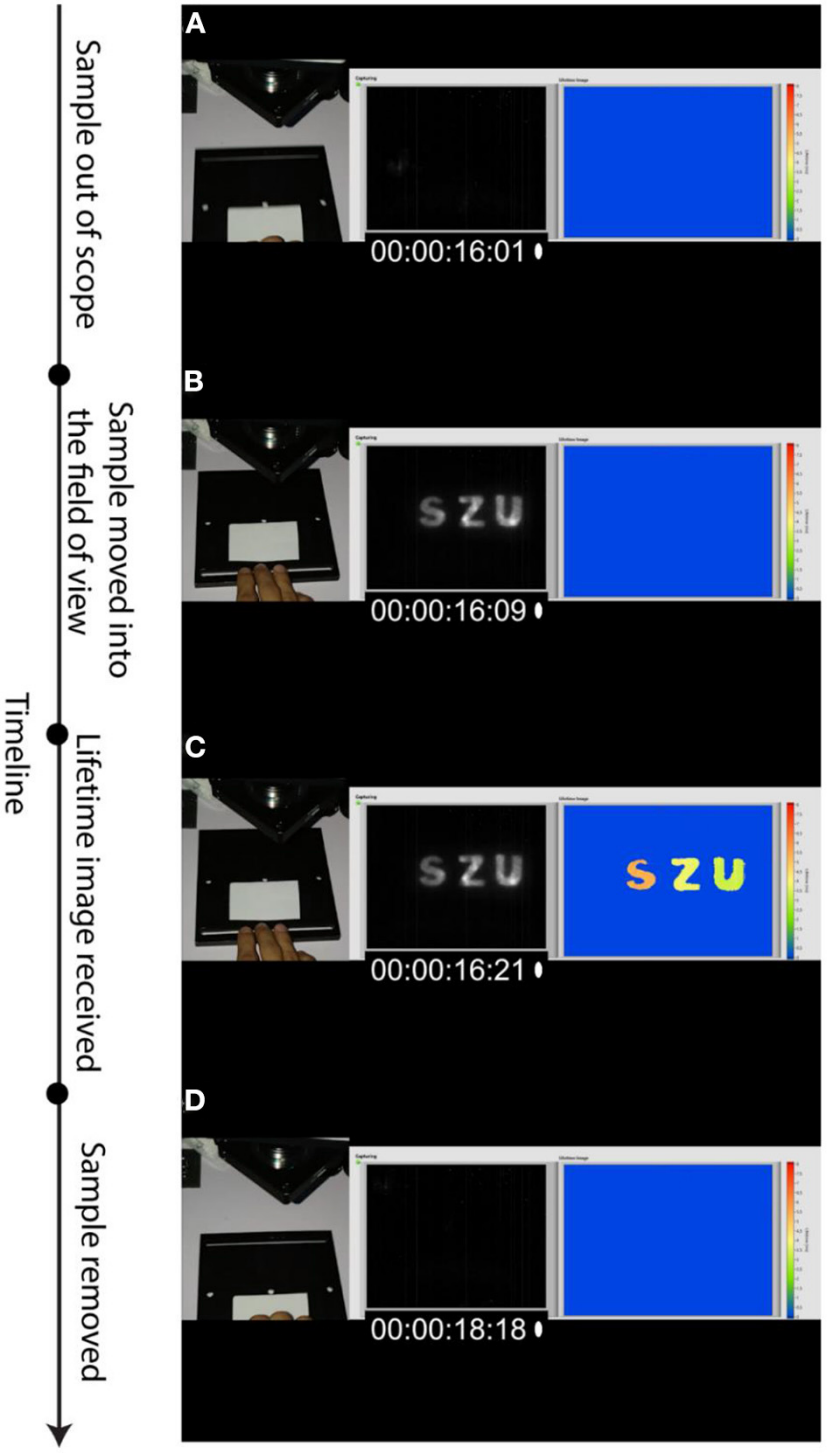
Timeline and major stages of RLD-assisted real-time decoding of SWIR PL pattern illustrated by the photographic images, selected video frames, and corresponding screens of the RLD-based software decoding lifetimes. Timecode (00:00:xx:yy) in every presented video frame shows seconds of the video (xx) and number of the frame within current second (yy). **(A)** The paper with invisible pattern is to be moved into the imaged area (time: 16 s, frame 1). **(B)** SWIR PL intensity image appearing when the pattern is moved into the imaging area (time: 16 s, frame 9). **(C)** Lifetime image generated after receiving two sequential time-gated images with different delays followed by RLD-based processing (time: 16 s, frame 21). **(D)** The paper with invisible pattern is moved out of the imaged area (time: 18 s, frame 18).

Finally, we have demonstrated the ability of the time-gated SWIR PL imaging setup and the RLD-based software, which process two PL intensity images of different delays to generate PL lifetime image, to working in real time. Two videos were acquired with this aim: (1) video of physical movement of the paper with the pattern in and out of the SWIR camera field of view (acquired by a cell phone camera); (2) computer screen recording with the LabVIEW-based software, which controls the SWIR camera, processes PL intensity images, and generates PL lifetime image using the RLD algorithm. These two videos were synchronized afterwards and four frames of each one were extracted and positioned next to each other to illustrate real-time PL lifetime imaging of anti-counterfeiting pattern. The stages of the anti-counterfeiting SWIR PL imaging, illustrated by combinations of the synchronized video frames, are presented in Figure 5. It shows the images when the patterned paper is about to be moved into the imaged area (Figure 5A), the pattern is moved into the SWIR camera field of view (imaged area), PL intensity image is acquired (Figure 5B), PL lifetime image is generated (Figure 5C), and the patterned paper is removed out of the imaged area (Figure 5D). The timecodes, which are seen in every selected video frame, are in a format of “hours:minutes:seconds:frames” (frames from 0 to 30 are counted within the current second). The total time between inserting patterned paper in the imaged area and removing it from there is <3 s, while PL lifetime image is seen to be generated in <1 s, as also confirmed by Supplementary Video 1.

## Discussion

We report on the real-time determination/imaging of SWIR PL lifetimes for anti-counterfeiting applications using time-gated SWIR PL imaging setup with pulsed laser diode as an excitation source, RENPs as anti-counterfeiting temporal tags, and an RLD method for fast decoding. It is worth noting that while the developed approach is applicable in broad temporal range (from tens and hundreds of microseconds to tens of milliseconds), the use of lifetimes of several milliseconds makes this method highly beneficial, due to combined advantages of simplicity, high speed, and accuracy. Throughout the conducted experiments with NaYF_4_:20%Yb^3+^, x%Er^3+^@NaYF_4_, the usability of such SWIR emitting RENPs as SWIR PL lifetime-coded tags for real-time anti-counterfeiting and security applications was shown. The Yb^3+^/Er^3+^ doped RENPs exhibit bright SWIR PL peaked at ~1,530 nm, with a lifetime that can be precisely tuned in a wide range by manipulation of Er^3+^ content, making these RENPs a highly promising material for anti-counterfeiting and security applications. Use of RLD algorithm allowed us to perform real-time imaging of PL lifetimes of RENPs both suspended in liquid and deposited on a paper. Three samples of RENPs with different SWIR PL lifetimes were clearly distinguished and lifetime values were determined in the post-processed images as well as in real-time running test with SWIR photoluminescent pattern.

It should be noted, however, that the use of the RLD method has its own limitations. First, it requires the PL decays to be monoexponential (Chan et al., 2001). In this regard, the employed RENPs are suitable as their PL decays can be well-fitted with a single exponent (Figure 2G). This also leads to an inability of RLD approach to separate (unmix) multiple signals in the single image pixel. This limitation can be addressed by utilization of dual lifetime determination (DLD) method, which can be considered as a modified version of RLD. In DLD, four PL images with different delay times are acquired and the RLD method is repeated twice for two different components in single pixel (two images to determine shorter component and two images for longer component) (Nagl et al., 2009), which results in longer data acquisition in comparison with simple RLD. However, DLD application is not needed when the PL in the imaged pattern is known to decay monoexponentially, as in our work. Another limitation of the RLD (and DLD) method is that the lifetime values are to be known preliminarily before the experiment (testing) in order to maximize the signal-to-noise ratio and, hence, the precision of the measurements. Nevertheless, this appears to be feasible in anti-counterfeiting applications.

In conclusion, our results open the pathway to real-time imaging of PL lifetimes for anti-counterfeiting applications. A combination of the PL time-gating system with RLD-based approach appears to be highly promising for applications in various fields, including information storage, real-time *in vivo* bioimaging, and particle tracking.

## Materials and Methods

### Synthesis of RENPs

The NaYF_4_: 20% Yb^3+^, x% Er^3+^ NPs (x = 0, 2, 20, 80) were prepared following the method previously reported (Li et al., 2017). First, RE-OA precursor was prepared using the following procedure. (0.8-x) mmol YCl_3_·6H_2_O, 0.2 mmol YbCl_3_·6H_2_O, x mmol ErCl_3_·6H_2_O, and 3 mmol sodium oleate were mixed with 3 ml of deionized water, 3.5 ml of absolute ethyl alcohol, and 7 ml of hexane, and the resulting mixture was heated at 60°C overnight. The organic phase solvent containing Ln-OA (Ln = Y, Yb, Er) was collected through a separatory funnel, and washed three times with deionized water in a separatory funnel. Second, the obtained Ln-OA precursor was mixed with 8 mmol sodium oleate, 5.2 ml of OA, 5.1 ml of OM, and 9 ml of ODE. The solution was then heated up to 100°C under argon gas protection with vigorous magnetic stirring for 60 min. Subsequently, 8 mmol solid ammonium fluoride was added to the solution and kept at 100°C for another 30 min. Last, the reaction mixture was heated to 300°C at a rate of 10 K·min^−1^, kept at this temperature for 30 min, and then allowed to cool down to room temperature naturally. The resulting products were precipitated by addition of 20 ml of ethanol, collected via centrifugation at 6,000 rpm for 10 min, washed twice with a 1:6 hexane/ethanol mixture, and finally dispersed in 10 ml of hexane for further use.

### Preparation of NaYF_4_: Yb, Er@ NaYF_4_ RENPs

The thermal decomposition method was utilized to coat the NaYF4 shell with a thickness of about 3 nm on NaYF_4_: Yb, Er core. Typically, 0.5 mmol Y_2_O_3_ dissolved in 10 ml of trifluoroacetic acid (TFA) aqueous solution with a concentration of 50%. The solution was heated at 90°C under stirring until the solution transformed into transparency, and then, the solution was evaporated to dryness under argon gas, yielding the shell precursor (CF_3_COO)_3_Y. Afterwards, 2 mmol of sodium trifluoroacetate (0.272 g) was added to (CF_3_COO)_3_Y precursor, together with 10 ml of OA, 10 ml of ODE, and 2.5 ml (0.25 mmol) of the pre-prepared NaYF_4_: 20% Yb^3+^, x% Er^3+^ NPs (x = 0, 2, 20, 50, 80); the mixture was then heated at 120°C for 30 min to distill low-boiling liquid, such as water and hexane. Then, the solution was heated to 300°C at a rate of 10 K·min^−1^ under argon protection. After keeping 30 min at 300°C, the reaction mixture was cooled to room temperature and the product was isolated as described above for the NaYF_4_: Yb^3+^, Er^3+^ core NPs.

### Measurements of PL Spectra and Lifetimes

UC and DS PL spectra in visible and NIR range were obtained using HORIBA Fluorolog-3 spectrofluorometer attached to an iHR320 spectrometer (Horiba) coupled with thermoelectrically cooled NIR-PMT detector (H10330B-75, Hamamatsu). As an excitation source, the fiber-coupled laser diode emitting at 975 nm (QSP-975-10, QPhotonics, USA) controlled by a laser driver (Arroyo Instruments, 4308-QCW) was employed: a collimated beam from the diode was introduced inside the sample chamber of the Fluorolog-3 and directed on the sample cuvette. Laser diode was used in CW mode for the PL spectra acquisition and in pulsed mode (100 μs pulse width) during PL lifetime measurements. In the latter case, signal from the output of the NIR PMT was recorded by the Digital Phosphor Oscilloscope (TDS 3034C, Tektronix) using a variable feed-through terminator (VT2, Thorlabs), and the PL decays were averaged over 128 excitation pulses. The iHR320 spectrometer was set to 1,000 and 1,530 nm for Yb^3+^ and Er^3+^, respectively. In addition, sync out signal from the laser driver was directly connected to an oscilloscope for external triggering. The experimentally obtained PL decays were fitted monoexponentially using Origin Pro software (OriginLab).

### Security Labeling

The imaging pattern was obtained by the direct deposition of RENP hexane suspension onto a commercially available printing paper, which was cut to form the “SZU.” Furthermore, those letters with deposited nanoparticles were placed between two sheets of the same white printing paper. This step allows our pattern to be invisible to the naked eye.

### SWIR PL Imaging

A home-built SWIR time-gated imaging system was developed based on a NIR-SWIR camera (Xeva-1.7-320, Xenics, Belgium), which is equipped with focusing optics (TEC-M55MPW, Computar, USA) and operates in 900–1,700 nm spectral range (Ziniuk et al., 2019). During imaging, the 1,200-nm long-pass filter (1200LP from Edmunds Optics.) was used in order to cut off Yb^3+^ emission and scattering from the exciting laser. A 975-nm fiber-coupled laser diode (QSP-975-10) powered by a QCW capable laser driver (Arroyo Instruments, 4308-QCW) was used as an excitation source, providing a power density of 30 mW/cm^2^ at the sample. Pulse modulation with 10-Hz repetition rate was performed in a burst mode in order to achieve one pulse per trigger received from the LabVIEW developed software. An InGaAs camera was synchronized with laser diode pulses using the “Sync out” output of the laser driver and triggered to capture image at precisely controlled delay times (10 μs to few seconds) and kept integrating for 10 ms. For the RLD, pairs of images with different delay times were obtained. For the image involving a single lifetime, the lifetime of each pixel can be determined by equation (Figure 1C). By synchronizing laser triggering, image acquisitions, and data processing, the process can be repeated, thus giving the real-time lifetime data as fast as four frames per second. All the mentioned operations were carried out using LabVIEW software.

## Data Availability Statement

The original contributions presented in the study are included in the article/Supplementary Material, further inquiries can be directed to the corresponding author/s.

## Author Contributions

RZ conceived the idea, developed the experimental setup and analyzing software, performed experiments, and drafted the manuscript. AY contributed to the development of the experimental setup and analyzing software. HL and GC synthesized the RENPs and characterized them with TEM. JQ supervised the project and revised and edited the manuscript. TO initiated the study, contributed to the idea development, supervised the project, and revised and edited the manuscript. All authors approved the final version of the manuscript.

## Conflict of Interest

The authors declare that the research was conducted in the absence of any commercial or financial relationships that could be construed as a potential conflict of interest.
